# Extensive Pyoderma Gangrenosum: A Challenging Diagnosis and Literature Review of Management

**DOI:** 10.7759/cureus.1486

**Published:** 2017-07-18

**Authors:** Faizan Yasin, Salman Assad, Mehr Zahid, Shuja A Malik

**Affiliations:** 1 Neurology, State University of New York at Buffalo; 2 Department of Medicine, Shifa International Hospital, Islamabad, Pakistan; 3 Internal Medicine, University of Lahore, Lahore, Pakistan; 4 Internal Medicine, Nawaz Sharif Medical College, University of Gujrat

**Keywords:** pyoderma gangrenosum, pathergy, corticosteroids, healing

## Abstract

Pyoderma gangrenosum is a very rare, non-infectious leukocytic dermatosis, which is often associated with an underlying systemic disease. It is usually diagnosed based on the apparent clinical findings and by excluding other causes of ulcerative skin diseases. Treatment modality includes the use of systemic steroids and oral steroids. Immunosuppressive agents, such as cyclosporine and mycophenolate mofetil, can also be added if it fails to respond to steroids. We report a case of pyoderma gangrenosum in an 85-year-old female who presented in the inpatient facility with rapidly enlarging necrotic, ulcerative lesions with accompanying fever. She was managed with systemic steroids to which she responded well. This led to the arrest of the initially progressive lesions with some residual scarring.

## Introduction

Pyoderma gangrenosum (PG) is a non-infectious leukocytic dermatosis with its classical ulcerative variant seen most commonly in affected patients. Several other clinical variants of PG also exist, which include parastomal, pustular, bullous, and granulomatous forms. The incidence of PG increases with age, seen more commonly in the fourth decade and onwards [1-2]. Females are more predisposed to developing this skin disease. Various comorbidities are found to have a bidirectional link with PG, in that not only does it predispose to those comorbidities like diabetes, peripheral vascular disease, and depression but itself may be caused by them [3]. PG exhibits the phenomenon of pathergy, which is the development or enhancement of skin lesions when it is subjected to trauma. Such is the case when debridement or any other surgical procedure is done on these lesions. Hence, such procedures should always be avoided. This also emphasizes the need to differentiate PG from other causes of ulcerative skin diseases and infectious processes. For this purpose, a skin biopsy is always required for its differentiation from other disease etiologies. The lesions are most commonly seen on legs. PG is often accompanied by fever, malaise, arthralgias, and myalgias. It is often associated with inflammatory bowel diseases (Crohn’s and ulcerative colitis) and rheumatoid arthritis [4]. Due to the potential involvement of other systems, a thorough workup, including complete blood count, chest x-ray, liver function tests (LFTs), biochemical profile, blood culture, and colonoscopy, might be needed.

## Case presentation

An 85-year-old female with no known comorbidities received from the emergency department presented to the inpatient department at Ittefaq Hospital (Trust) in Lahore, Pakistan with a seven-day history of fever and multiple painful lesions/wounds on both of her legs, feet, and left arm. The patient revealed that these lesions have been existent for four weeks. First, they appeared as multiple purpura and blisters that later ruptured, leading to raw areas and then necrotic ulcerated lesions with thick brown crusting. Just 11 days earlier, she had presented to the dermatology outpatient clinic with the complaint of fever and fluid-filled blisters, relatively smaller in size. In that visit, she was diagnosed as a case of vasculitis, likely microscopic polyangiitis or fixed drug eruption on the basis of clinical findings. Further workup was required for a definitive diagnosis due to which various tests, including complete blood count (CBC), hemoglobin A1c (HbA1c), liver function tests (LFTs), serum creatinine, and a follow-up visit in one week time, were advised. She was prescribed Augmentin (amoxicillin clavulanate), 625 mg orally three times daily; paracetamol orally three times daily; cetirizine, 1 mg orally one tablet at night; prednisolone, 5 mg orally two tablets after breakfast; omeprazole, 20 mg orally one capsule before breakfast; and bacitracin/polymyxin/neomycin/lidocaine - triple antibiotic first aid ointment topically twice daily for seven days. Besides, she was advised to apply calamine lotion, cover the lesions with pyodine dressing, and change them daily for seven days. 

**Figure 1:**
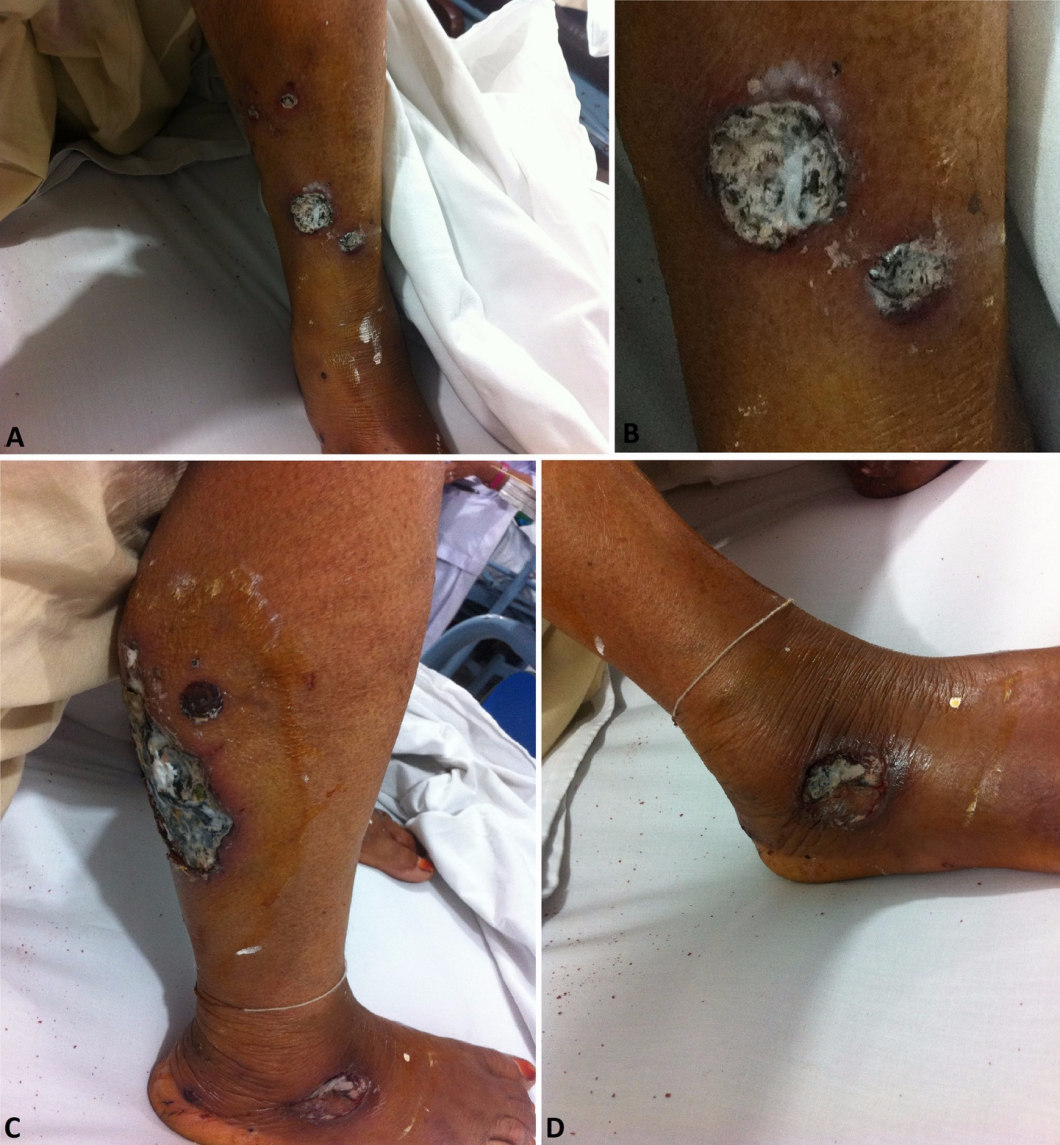
Clinical findings (A, B) Ulcers having a purulent base, asymmetrical borders, and erythematous surrounding. (C) A large ulcerative lesion having a purulent base with purple raised borders and erythematous surrounding. (D) Ulcer base covered with granulation tissue. The top layer of skin missing

**Figure 2:**
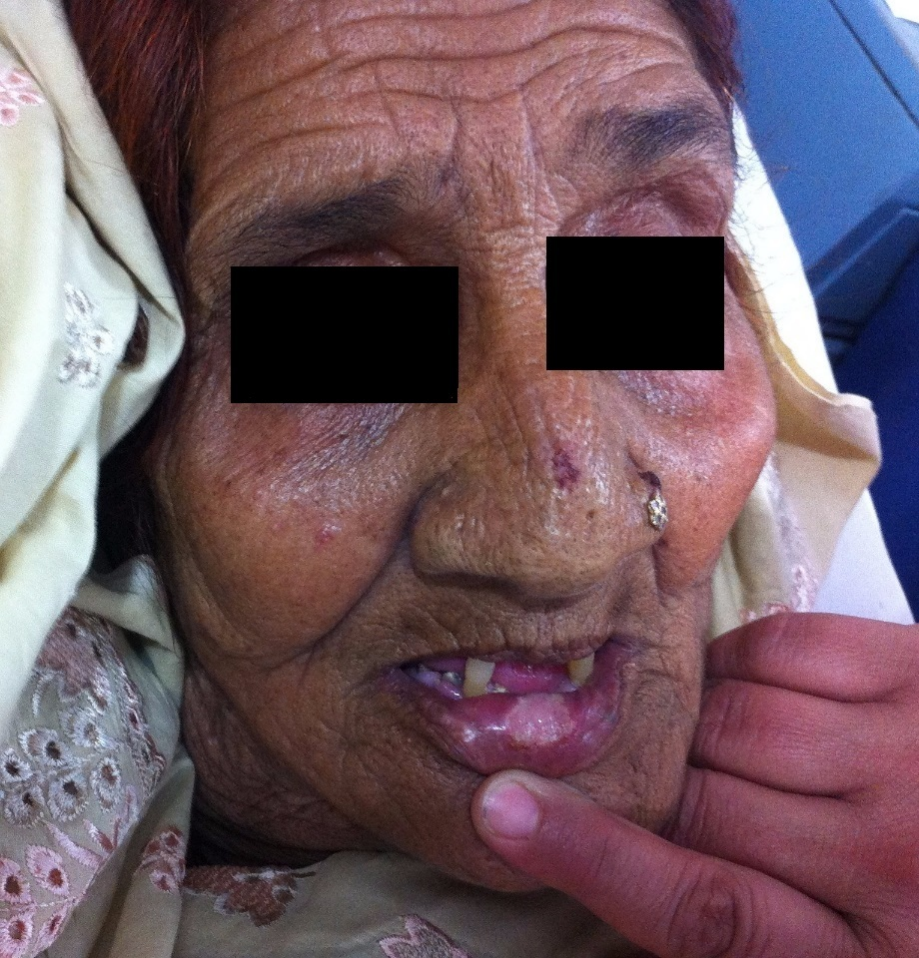
Aphthous ulcer on the lower labial mucosa

On her recent admission, she also complained of having oral ulcers and easily scraped off-white patches on the tongue, accompanied by painful and difficult swallowing. Her fever was high-grade, associated with chills, and was continuous. She denied any history of nausea, vomiting, or urinary or bowel complaints. There was no significant past medical history or known allergy. Examination of the lower extremities revealed multiple wounds with necrosis and a dirty base (Figure 1A-D). There was pallor in the conjunctiva, mucositis (Figure 2), and white patches in the oral cavity, likely oral thrush. The dorsalis pedis pulse was palpable in both feet and there was no evidence of pedal edema. Similar lesions were also present on left arm; however; they were lesser in number. Rest of the examination was unremarkable with normal heart sounds, normal vesicular breathing, soft non-tender abdomen, positive bowel sounds, no visible jaundice, and normal neurologic exam. She was diagnosed as a case of vasculitis/pyoderma gangrenosum, along with aphthous ulcers and oral candidiasis.

Her treatment began with oral nystatin drops four times daily; miconazole gel, local application four times daily; IV Penetrex (enoxacin), a broad-spectrum fluoroquinolone antibacterial agent, 4.5 g three times daily; paracetamol, 500 mg two tablets if needed; cetirizine, 10 mg orally at night; calamine lotion local application three times daily; Fucidin®, a topical antibiotic ointment, local application three times daily; IV tramadol, 25 mg diluted in 20 cc normal saline twice daily; Vitamin D3 injection once weekly; IV hydrocortisone sodium succinate, 100 mg three times daily; prednisolone, 5 mg orally three times daily; linezolid, 400 mg orally twice daily; omeprazole, 40 mg orally twice daily; and diclofenac sodium, 50 mg twice daily.

An extensive serologic evaluation was completed, which included the rheumatoid factor, antinuclear antibody, and anti-double-stranded (ds) deoxyribonucleic acid (DNA). Erythrocyte sedimentation rate (ESR), C-reactive protein (CRP), serum electrolytes, renal function tests, serum urea, creatinine, and liver function tests were also ordered (Tables 1-3). A hepatitis panel was ordered due to its causal relationship with pyoderma gangrenosum but was non-contributory. Laboratory investigation showed an inflammatory reaction and anemia. An underlying infection was indicated by the raised white blood cell (WBC) count with an elevation of neutrophils on the differential count. Platelets were slightly elevated, along with a raised alkaline phosphatase (ALP) and gamma-glutamyl transferase (GGT). A chest x-ray posteroanterior (PA) view was performed, which was normal. She also underwent evaluation by numerous other specialists, including a dermatologist, gastroenterologist, and vascular surgeon. The prothrombin time (PT) and activated partial thromboplastin time (aPTT) were ordered the next day and came out normal (Table 4). A complete blood count (CBC) with differential count was repeated, which revealed a raised WBC count with an elevation of neutrophils (Table 5). Serum alanine aminotransferase (ALT), serum creatinine, and hemoglobin A1c (HbA1c) showed normal values (Table 6).

**Table 1:** Complete Blood Count (CBC) with Differential WBC: white blood cell; RBC: red blood cell; HCT: hematocrit; MCV: mean corpuscular volume; MCH: mean corpuscular hemoglobin; MCHC: mean corpuscular hemoglobin concentration; MPV: mean platelet volume; RDW: red cell distribution width

Test(s)	Result	Normal Value	Unit
BLOOD COMPLETE PICTURE			
WBC Count	14.52	4 - 11	10^3/uL
RBC Count	3.65	4 - 5.2	10^6/uL
Hemoglobin	10.7	11.5 - 16	g/dL
HCT	31.1	33 - 45	%
MCV	85.2	79 - 95	fL
MCH	29.3	26 - 32	pg
MCHC	34.4	32 - 36	g/dL
Platelets	468	150 - 450	10^3/uL
MPV	10.9	7.2 - 13	fL
RDW	14.2	11.5 - 14.5	%
DIFFERENTIAL COUNT			
Neutrophils	85	34 - 70	%
Lymphocytes	10	19 - 52	%
Monocytes	3	2 - 12	%
Eosinophils	2	1 - 6	%

**Table 2:** Further Laboratory Investigations

Test(s)	Result	Normal Value	Unit
Blood Urea Nitrogen (BUN)	47	10 - 50	mg/dL
Serum Creatinine	0.7	0.5 - 0.9	mg/dL
Blood Sugar (Random)	114	< 140	mg/dL
Sodium (Na)	133	132 - 146	mmol/L
Potassium	4.3	3.3 - 5.1	mmol/L
Liver Function Tests			
Total Bilirubin	0.5	0.1 - 1.0	mg/dL
Alanine aminotransferase (ALT)	34	5 - 31	u/L
Aspartate aminotransferase (AST)	16	5 - 32	u/L
Alkaline Phosphatase (ALP)	196	35 - 104	u/L
Serum Total Protein	6.1	6.4 - 8.3	g/dL
Serum Albumin	2.0	3.4 - 4.8	g/dL
Gamma Glutamyl Transferase (GGT)	103	7 - 32	u/L

**Table 3:** Inflammatory Markers

Test(s)	Result	Normal Value
Erythrocyte Sedimentation Rate (ESR)	91	0 - 20
C-reactive protein (CRP)	192	< 5
Rheumatoid Factor (RF)	5	< 30 IU/mL – Negative 30 - 50 IU/mL – Weak Positive > 50 IU/mL – Positive
Anti-nuclear antibody (ANA)	Negative	Negative

**Table 4:** Coagulation Profile

Test(s)	Result	Normal Value	Unit
Prothrombin Time (PT)			
Patient Value	11.7	9 - 14	SEC
Control Value	10.2		SEC
International Normalized Ratio (INR)	1.15		
Activated Partial Thromboplastin Time (APTT)			
Patient Value	28.0	22 - 31	SEC
Control Value	26.4		SEC

**Table 5:** Complete Blood Count (CBC) with Differential RDW: red cell distribution width WBC: white blood cell; RBC: red blood cell; HCT: hematocrit; MCV: mean corpuscular volume; MCH: mean corpuscular hemoglobin; MCHC: mean corpuscular hemoglobin concentration; RDW: red cell distribution width

Test(s)	Result	Normal Value	Unit
BLOOD COMPLETE PICTURE			
WBC Count	17	4 - 11	10^3/uL
RBC Count	4.04	4 - 5.2	10^6/uL
Hemoglobin	11.7	11.5 - 16	g/dL
HCT	36	33 - 45	%
MCV	89	79 - 95	fL
MCH	29	26 - 32	pg
MCHC	32	32 - 36	g/dL
Platelets	415	150 - 450	10^3/uL
RDW	12.2	11.5 - 14.5	%
DIFFERENTIAL COUNT			
Neutrophils	80	34 - 70	%
Lymphocytes	14	19 - 52	%
Monocytes	4	2 - 12	%
Eosinophils	2	1 - 6	%

**Table 6:** Laboratory Findings

Test(s)	Result	Normal Value	Unit
Alanine Aminotransferase (ALT)	21	5 - 31	u/L
Serum Creatinine	0.7	0.5 - 0.9	mg/dL
Glycosylated Hemoglobin (HbA1c)	5.7	4.8 - 5.9	%
Interpretation	Non-Diabetic: < 5.9% Prediabetic: 5.9 – 6.4% Diabetic: >/= 6.5%		

The patient had poor oral hygiene. She later complained of having insomnia due to worsening of pain in the wounds. Fever spikes of 102 F and 100 F occurred on the fourth day of admission, which was later well controlled with medications. Surgical consultation was made and a color Doppler study of both lower limbs was done. Mild mural calcific atherosclerotic changes were noted in the popliteal and tibial arteries without causing significant stenosis, as good velocity triphasic flow pattern was seen into the foot. No critical stenosis or occlusion was noted. Iliac, femoral, popliteal, anterior tibial, and posterior tibial veins were also patent. Veins showed normal blood flow and caliber. They also showed normal compressibility. Normal phasic changes of flow with breathing were present. Hence, there was no evidence of deep vein thrombosis. The only mild peripheral arterial atherosclerotic disease was found without causing any stenosis.

A deep elliptical skin biopsy, including skin and subcutaneous tissue, was obtained under local anesthesia and sent for histopathological examination. A skin biopsy specimen obtained from a lesion of the left lower leg, a skin -covered fragment, which measured 2.0 x 1.0 x 0.5 cm. A note was made of the gangrenous wounds on the calf and debridement was strictly avoided due to the risk of pathergy. Intravenous normal saline was given, along with the application of a pyodine solution, and Fucidin cream-topical antibiotic ointment was applied to the lesions. The patient was mobilized with help. SpO2 was 98%, blood pressure (BP) was 100/70 mm Hg, respiratory rate (RR) 18/min, and pulse 102/min. Intake was 350 ml, whereas urinary output was 600 ml. The patient had not passed stool.

The patient’s condition improved markedly over the next few days. Fever and pain associated with the lesions had subsided. Oral candidiasis had resolved with the use of oral nystatin. Analgesic muscle relaxant (paracetamol and orphenadrine citrate combination) orally and IV ketorolac, 30 mg, were given for pain relief on what turned out to be her last day of hospital stay. The patient was then discharged from the hospital and a follow-up visit was scheduled in two week's time. She was prescribed multiple medications on discharge, including linezolid, 600 mg orally twice daily; cetirizine, 10 mg one tablet at night; analgesic muscle relaxants (paracetamol and orphenadrine citrate combination) three times daily; Fucidin cream topical application three times daily; Tramadol, 50 mg orally twice daily; prednisolone, 5 mg orally three times daily; and Vitamin D3 injection once weekly for mild bony aches and muscle weakness that patient had complained about.

**Figure 3:**
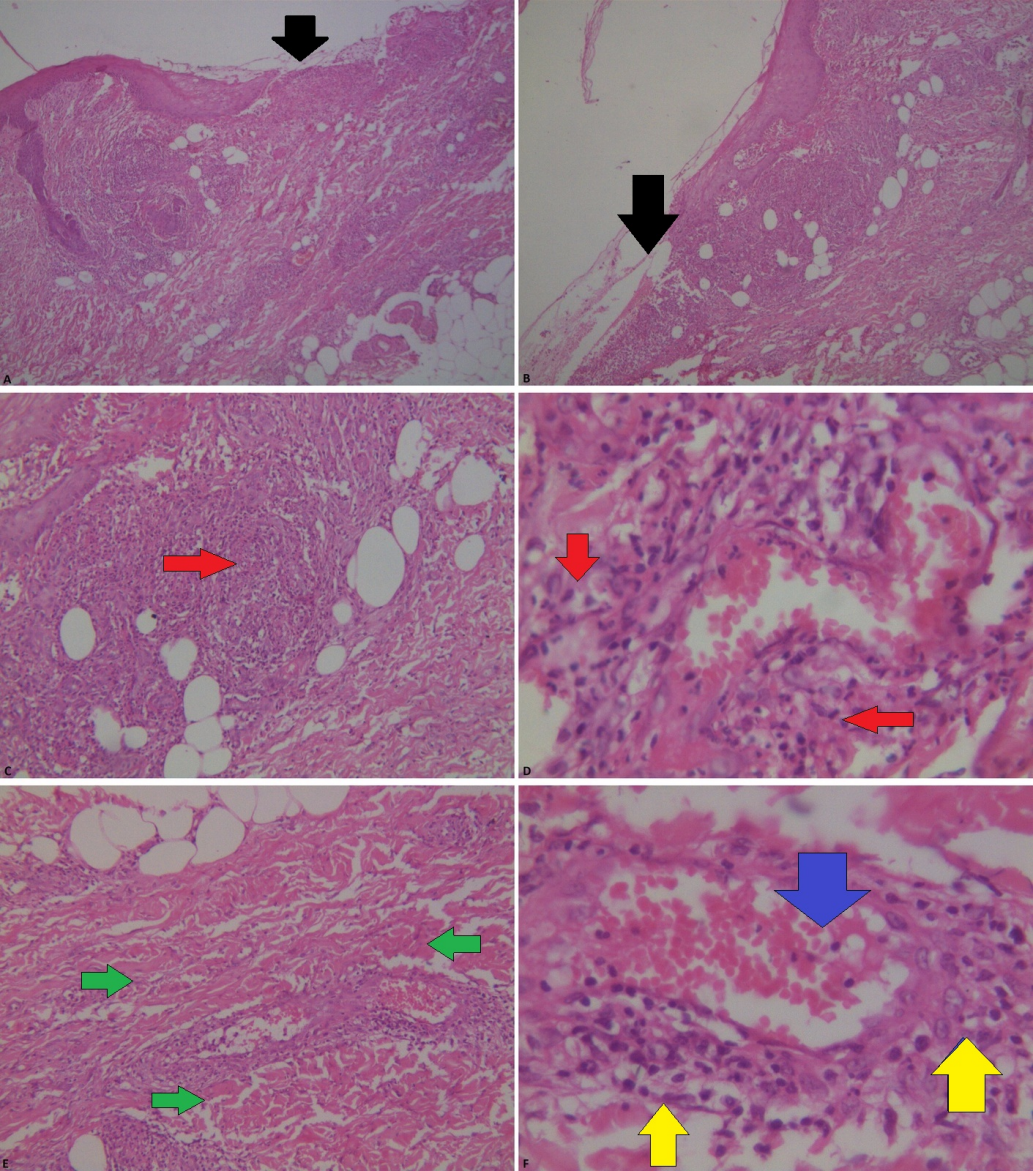
Histopathology (A, B) Surface ulceration. (C, D) The adjacent dermis shows acute and chronic inflammation. Neutrophilic infiltrate (red arrows) indicates acute inflammation, plus neovascularization seen (blue arrow). (E, F) Fibroblast proliferation (green arrow). Lymphocytic infiltrate indicating chronic inflammation (yellow arrows)

Biopsy results came out a week later. Gross examination revealed an area of ulceration on the skin, which measured 1.0 x 8.0 cm, whereas microscopic examination revealed skin lined by benign keratinizing stratified squamous epithelium and showed surface ulceration with underlying microabscess formation. The inflammatory cells extended into the underlying subcutaneous fat. The adjacent dermis showed acute and chronic inflammation (Figure 3 A-F). A few areas showed leukocytoclastic vasculitis. No granuloma or malignancy was seen. All these findings were consistent with pyoderma gangrenosum.

**Figure 4:**
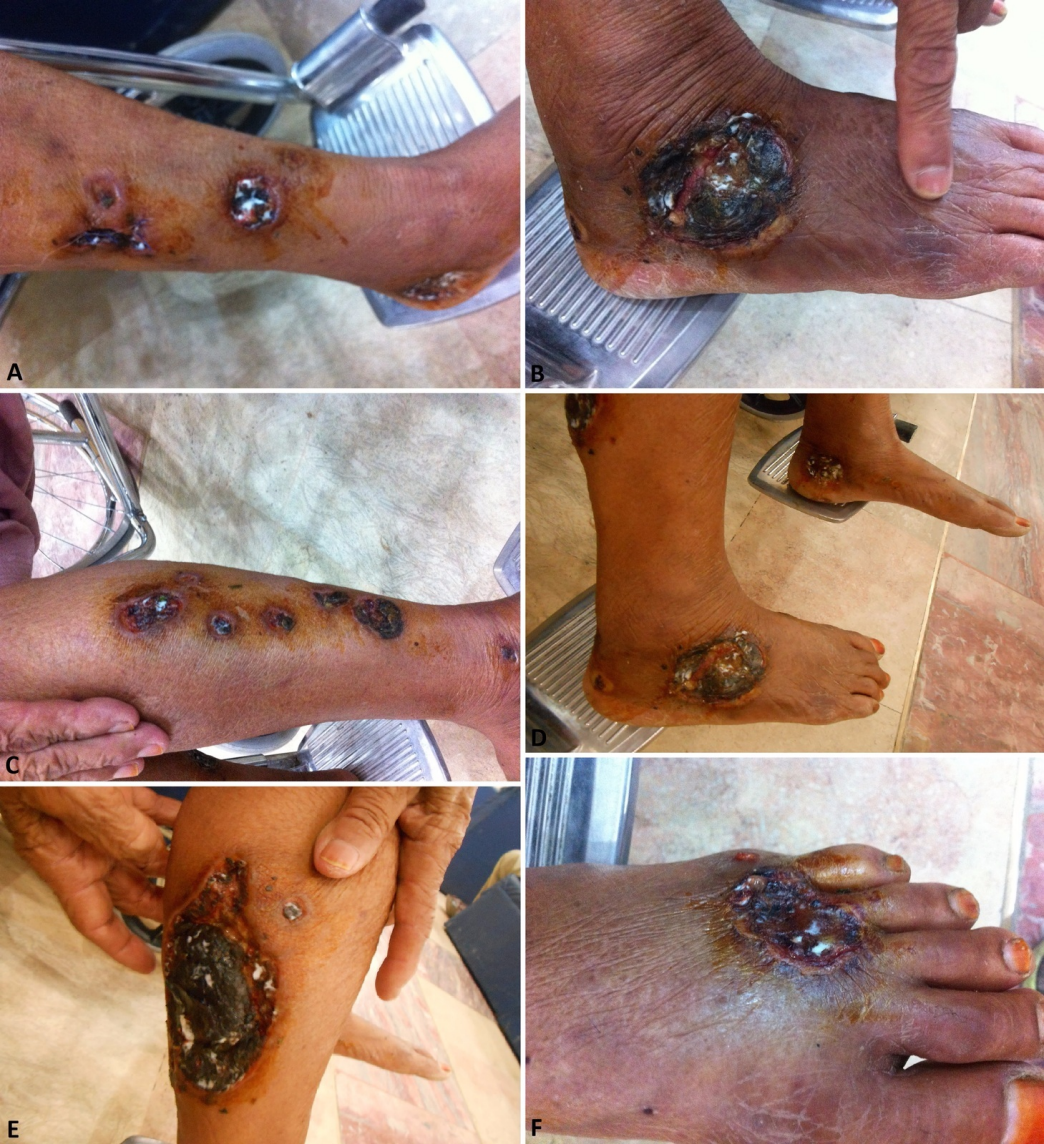
Ulcers with asymmetrical borders, residual scarring and necrosis. Base of ulcers covered with granulation tissue

The patient visited the Medical Outpatient Department (OPD) two weeks later. She was apparently in much better condition. There was no complaint of fever or pain. The lesions showed residual scarring (Figure 4A-F). Treatment was successful in arresting the process, but complete healing may take months. This may also be attributed, in part, to the mild peripheral arterial atherosclerotic disease. She was referred to surgical OPD, where pyodine dressings were done on the lesions. Subsequent visits, every second week, were also advised for the same purpose. 

## Discussion

PG is a very challenging diagnosis and differentiating it from other ulcerative skin diseases is an essential component in its appropriate management. As mentioned previously, pathergy phenomenon could eventually lead to a worse prognosis for the patient. The classic variant of PG usually presents as small red-blue papules that then transform into painful ulcerative lesions with erythematous-violaceous edges. This was exactly the case in our case presentation. Systemic and oral steroids play an essential role in arresting the process. Resolution of the lesions is almost never seen; however, arresting the process of progression is the mainstay of management in PG [5]. The same was the case in our patient; her lesions had been arrested, showed healing and residual scarring. Diagnosing this condition properly is very important. The major diagnostic criteria for the classic variant of PG includes rapidly progressing ulcerative, necrotic skin lesions with violaceous, irregular edges and exclusion of other ulcerative skin diseases. In addition to that, two minor criteria must also be met. These include pathergy, rapid response to corticosteroids, underlying systemic diseases like seronegative spondyloarthropathies known to be associated with PG (i.e., inflammatory bowel diseases, hematological malignancies, and arthritis), and histopathology showing neutrophilic infiltrates, with or without lymphocytic vasculitis [6]. Our patient did meet the above criteria.

## Conclusions

A multidisciplinary approach is essential in managing this complex condition as was the case in our hospital setting, where the patient underwent evaluation by numerous specialists, including a dermatologist, gastroenterologist, and vascular surgeon. This led to a satisfactory outcome for the patient. Treatment was successful in arresting the process, but complete healing may take months. This may also be attributed, in part, to the mild peripheral arterial atherosclerotic disease in this patient. Misdiagnosing the condition can lead to inappropriate management and can worsen the condition. Hence, the need for referral to a dermatologist is essential as was seen in the present case of PG where debridement was strictly prohibited on the basis of her clinical findings.
